# Airway Epithelial Cell Cilia and Obstructive Lung Disease

**DOI:** 10.3390/cells5040040

**Published:** 2016-11-11

**Authors:** Asma Yaghi, Myrna B. Dolovich

**Affiliations:** 1 Firestone Research Aerosol Laboratory, Fontbonne Bldg. Room F132, Hamilton, ON L8N 4A6, Canada; yaghia@primus.ca; 2 Department of Medicine, McMaster University, Hamilton, ON L8S 4L8, Canada; 3 St. Joseph’s Healthcare, Firestone Institute for Respiratory Health, 50 Charlton Ave East, FIRH Room T2135, Hamilton, ON L8N 4A6, Canada

**Keywords:** epithelium, lung disease, ciliary function, COPD, inflammation, cilia

## Abstract

Airway epithelium is the first line of defense against exposure of the airway and lung to various inflammatory stimuli. Ciliary beating of airway epithelial cells constitutes an important part of the mucociliary transport apparatus. To be effective in transporting secretions out of the lung, the mucociliary transport apparatus must exhibit a cohesive beating of all ciliated epithelial cells that line the upper and lower respiratory tract. Cilia function can be modulated by exposures to endogenous and exogenous factors and by the viscosity of the mucus lining the epithelium. Cilia function is impaired in lung diseases such as COPD and asthma, and pharmacologic agents can modulate cilia function and mucus viscosity. Cilia beating is reduced in COPD, however, more research is needed to determine the structural-functional regulation of ciliary beating via all signaling pathways and how this might relate to the initiation or progression of obstructive lung diseases. Additionally, genotypes and how these can influence phenotypes and epithelial cell cilia function and structure should be taken into consideration in future investigations.

## 1. Introduction

The roles of cilia in development and disease are vast [1]. In the lung, cilia are tiny hair-like structures that move mucus and debris up the respiratory escalator. Identification of cilia in relation to lung disease goes back to the early 1900s. Kartagner recognized (1933) the clinical triad of situs inversus, chronic sinusitis, and bronchiectasis now known as Kartagner’s syndrome (KS). KS is inherited via an autosomal recessive pattern with symptoms resulting from defective cilia motility. However, KS is only part of a larger group of disorders known as primary ciliary dyskinesia (PCD) to differentiate them from the acquired types [2,3,4,5,6,7]. Ciliary beating of airway epithelial cells constitutes an important part of the mucociliary transport apparatus [8,9,10]. To be effective in transporting secretions out of the lung, the mucociliary transport apparatus must exhibit a cohesive beating of all ciliated epithelial cells that line the upper and lower respiratory tract [11]. Similar beat frequencies have been observed in nasal, tracheal, and bronchial epithelial cells [8,9,12]. Structure-function studies have helped identify the ciliary targets that participate in regulating ciliary beating, however, our understanding of such regulation is far from complete [3,13,14,15]. Many endogenous and exogenous factors can influence cilia function [16,17,18,19,20,21] with efficient mucociliary transport relying on effective regulation of ciliary beating [11,22]. The ultrastructure of cilia is well documented [23,24]. Yet little is known about the role of cilia in inflammatory and obstructive lung diseases [15] despite correlations between ciliary structure and ciliary function [4,22,25,26,27]. Recent studies of neonates and children with respiratory distress outlined clinical features of neonatal and childhood PCD and their associations with ultrastructural defects and genotype [28,29]. Additionally, links between genetics and ciliary function and structure are demonstrated in several studies [30,31,32,33]. For information on the relationship between genes and cilia structure and function, we refer the readers to recent extensive reviews on this subject [1,3,15,31,34,35]. Another important factor in the function of cilia is the mucus layer composition and thickness [36,37,38]. In this review, we will summarize what we know so far about the respiratory epithelium and specifically airway epithelial cell cilia and their role in inflammatory lung diseases specifically Chronic Obstructive Pulmonary Disease (COPD) and asthma. More research is required to determine the structural-functional regulation of ciliary beating via all signaling pathways and how this might relate to the initiation or progression of lung diseases such as COPD and asthma.

## 2. Airway Epithelium: A Specialized Physical, Secretory, and Regulatory Barrier

Airway epithelium is the first line of defense against exposure of the airway and lung to inflammatory stimuli and antigens. Epithelial cells form a physical barrier to bacteria and viruses, allergens, dust particles, and air pollutants. In addition, the airway epithelial cells provide an antimicrobial function via production of mucus, immunoglobulins, and defensins. The airway epithelium plays a regulatory role and a pro-inflammatory role through the release of neuropeptide degrading enzymes, endothelin, nitric oxide, TGF-β, arachidonic acid metabolites, and cytokines [22,39,40,41,42,43]. Epithelial cells line the airways form the nasal cavity down to the alveoli. A variety of cells make up the epithelium: the basal cells, which are the stem or progenitor cells for the epithelium and differentiate to form the other cells in injury and repair; the ciliated cells, which provide the mechanism for moving the mucus blanket and have also been reported to be involved in epithelial cell trans-differentiation and repair [44]; the goblet cells, which secrete the mucus; and the brush cells, which are involved in drug metabolism [45]. 50%–80% of epithelial cells lining the airways are ciliated; therefore they are the predominant cell type within the human airways [45] and specifically the smaller airways [46]. This contrasts to rodents where less cilia line the smaller compared to the larger airways [47]. The liquid periciliary layer (PCL) and the mucus layer on top of the PCL layer also line the airway epithelium from the upper regions of the lung to the alveoli, this layer gets thinner as the airways become smaller in diameter until the thickness is <0.1 μm in the alveoli [45]. Therefore, the airway epithelium is a specialized barrier with multiple functions. Any modifications of this barrier or its components can lead to lung disease.

The ciliated airway epithelium: The role of the cilia in traits (inflammation, exacerbation), symptoms (sputum production and cough), and progression of lung disease (relation to disease severity) is not fully understood. Cilia features that may contribute to lung disease include cilia function (ciliary beat frequency-CBF- and wave pattern), the length of the cilia, the ratio of ciliated to non-ciliated areas, cilia structure, and the susceptibility of the cilia to endogenous and exogenous factors that modify their CBF and quality of motion [11,13,18,20,22,24,48,49,50,51,52,53]. Random ciliary orientation has also been reported as a possible cause of respiratory disease [54,55].

**Figure 1:**
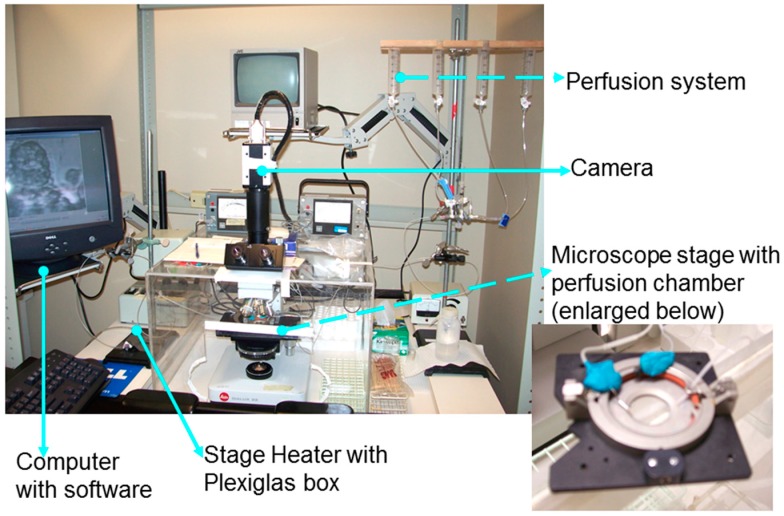
High Speed Digital Imaging Setup for data acquisition. The Motion Analyzer System attached to the microscope (MotionScope 1000 S monochrome, MotionScope PCI High Speed Digital Imaging System from Redlake MASD Inc., San Diego, CA, USA). A ciliated Epithelium specimen is placed in a perfusion chamber (Dvorak-Stotler Controlled Environment Culture System, Nicholson Precision Instruments Inc., Bethesda, MD, USA) attached to a gravity-fed perfusion system which delivers perfusate at the rate of 0.25 mL/min. The chamber is placed under the phase contrast microscope (E. Leitz, Wetzlar, Germany). A Plexiglas enclosure and an air stream stage heater (Nicholson Precision Instruments Inc., Bethesda, MD, USA) maintain the 37 °C temperature of the preparation. A MotionScope high-speed digital camera and PCI application software, running in a Windows environment (Redlake MASD Inc.), is used for image acquisition. A video segment is recorded for each area. Each video segment is stored in a file for later retrieval and analysis (Figure 2).

**Figure 2:**
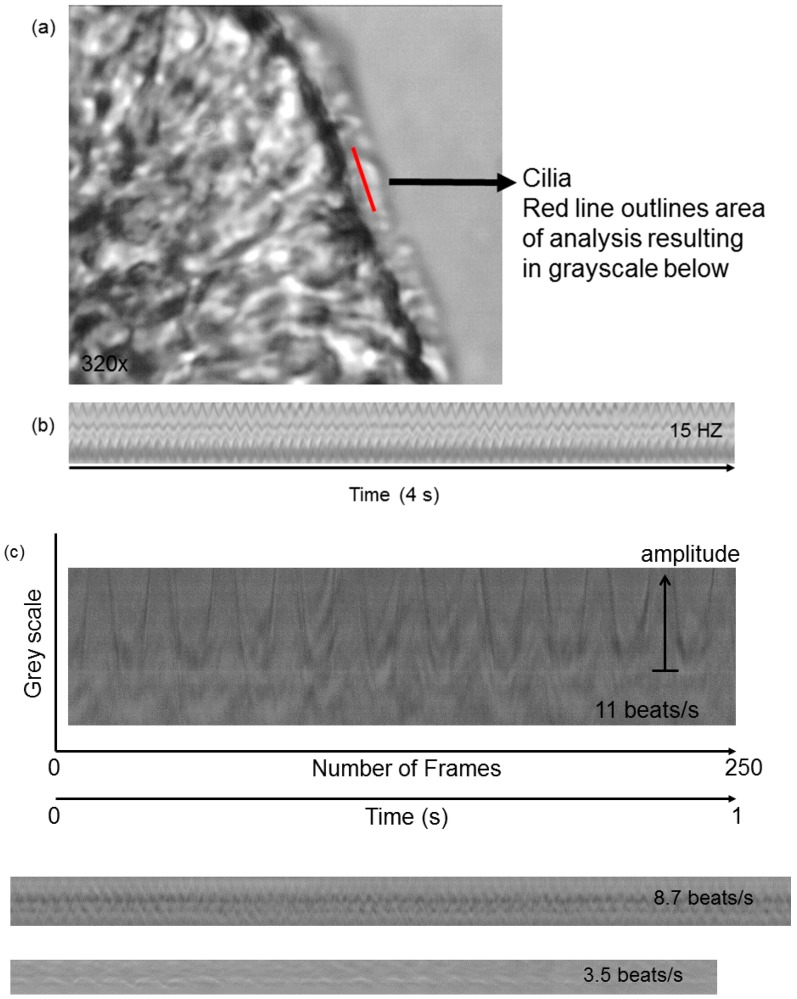
Video analysis and calculation of CBF in a nasal epithelial strip of cells. (**a**) Cilia are outlined; (**b**) Grayscale of beating cilia expressed per time and indicating 15 beats/s (i.e., Hz). Magnification 320×; (**c**) Ciliary beat frequency patterns illustrating amplitude and beat patterns. The video recordings are obtained and analyzed using ProAnalyst video imaging processing software (XCitex, Cambridge, MA, USA). Briefly, areas on the edge of the epithelium with beating cilia are analyzed using the one-dimensional tracking algorithm included in the motion analysis software ProAnalyst. A line is drawn across a segment of cilia (Figure 2a). The software captures the motion history within the analysis grid for the duration of the video and records the gray-scale intensity variation as a function of time (Figure 2b,c). The resulting files are calibrated as pixels/unit. This tracking process allows the plotting of the sinusoidal waveform generated by the beating cilia and the determination of the frequency (Hz). Figure 2c Evaluation of ciliary function (CBF and waveform) shows a 1 s CBF recording of 11 beats/s and CBF waveform panels of 8.7 beats/s and 3.5 beats/s. The resolution of the obtained images at a microscope magnification of 320× and a sampling rate of 250 frames per second is 480 × 420 pixels, where one pixel corresponds to 0.158 μm.

**Figure 3:**
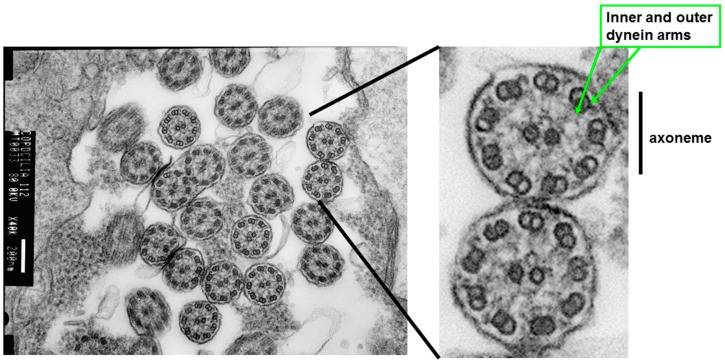
Ultrastructure of airway cilia. An illustration of Transmission Electron Microscopy (TEM) demonstrating axonemes. TEM sections of airway cilia can be analyzed for the number of inner and outer dynein arms, central tubules, and orientation of cilia.

Airway epithelial cell cilia are motile cilia, but these cilia have sensory functions and can respond to mechanical and chemical stimuli [56,57,58,59]. In bronchial explants of human respiratory mucosa, ciliary activity was significantly influenced by chemical (pH) and physical properties (viscosity) of the liquid medium in which the cilia beat [60]. In our studies, we demonstrated that tonicity and viscosity of the medium bathing human bronchial cilia can modify CBF [53]. Cilia are 6–7 µm long and 0.2–0.3 µm in diameter. Airway cilia move in a coordinated metachronous beat pattern moving mucus up the airway tree. A range of CBF of 12–16 Hz has been reported in nasal cilia of healthy subjects [61,62,63,64]. We have observed that cilia from nasal epithelial cells of healthy volunteers beat with a frequency of 14.2 ± 1.0 Hz (*n* = 10, ages 22–52 years) at 37 °C and exhibit the same metachronous beat pattern (laboratory data). Each beat constitutes of an effective and recovery stroke, and a rest phase which influence the amplitude, CBF and beat pattern [65]. Figure 1 shows a high-speed imaging setup for data acquisition of cilia function. Figure 2 illustrates how ciliary function can be evaluated by recording CBF and beat patterns. In terms of structure, cilia are microtubule-based cell organelles extending from the basal body, a centriole at the apical cell surface, containing axonemes. An axoneme is the microtubule cytoskeleton of the cilium, consisting of a ring of nine doublet microtubules surrounding a central pair (9+2). Inner and outer dynein arms (Figure 3) of each microtubule doublet generate the force needed for motility in an ATP-dependent process [24,65]. Inner dynein arms or radial spoke defects correlate with problems in ciliary bending, and outer dynein arms defects correlate with a decline in CBF, while the absence of the central microtubule pair has been correlated with a circular beat pattern [4,7,22,25,26,27]. The cilia are dynamic organelles. Functional and structural components of cilia are synthesized in the cytoplasm and these are transported up and down the cilium by a specialized system known as the intraflagellar transport system. Many ciliary proteins have been identified, yet their exact role in the cell remains unknown [14,66,67,68]. Cilia membranes are contiguous with the plasma membrane of the cell and contain specific receptors and ion channel proteins that initiate signalling pathways controlling motility, mechanical or chemical stimuli [13,24,56]. CBF in airway epithelial cells depends on the cyclic nucleotides cAMP and cGMP and intracellular calcium concentration [69,70,71,72]. In human airway epithelia, sAC is localized to motile airway cilia and it contributes to the regulation of human airway CBF [73]. Changes in epithelial cell intracellular pH [74] and intracellular bicarbonate are additional factors that might influence CBF through direct effects on dynein arms. The structural-functional regulation of CBF via many signalling pathways has been reviewed [13], yet a lot of research is required to determine how this regulation might influence the initiation or progression of obstructive lung diseases.

## 3. What Modulates Airway Cilia Function?

Ciliary beat frequency (CBF) and wave pattern are important measures of epithelial cell function and these can be modified by various exogenous and endogenous mediators. In 1981, Rossman and co-investigators observed that some patients with KS had motile cilia with abnormal ciliary structure and uncoordinated beating and used the term PCD which is now used to describe all congenital cilia disorders [4,7]. However, when structural cilia defects are not identified, cilia function can still be impaired by various exposures that can affect cilia function directly or can modify the secretions and viscosity of the mucus layer which is transported up the respiratory escalator by cilia [20,38,52,53,75].

**Figure 4:**
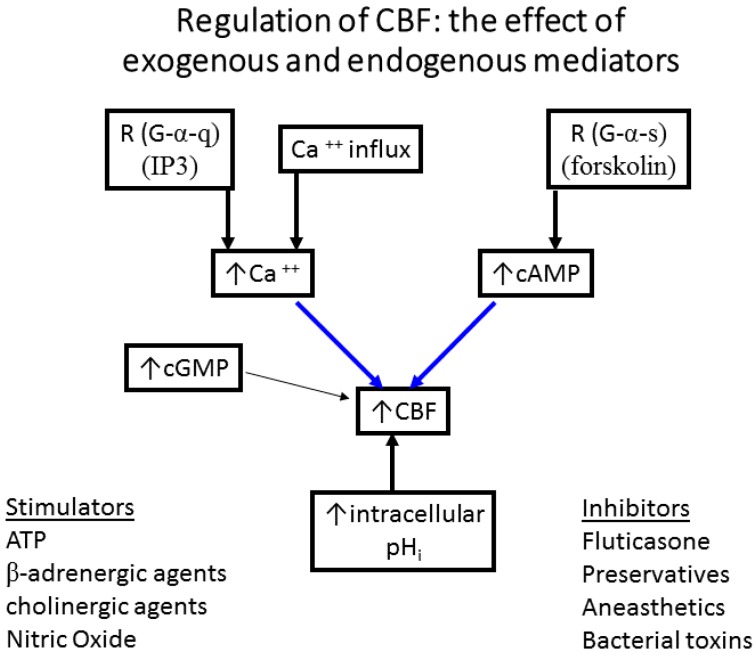
The effect of exogenous and endogenous mediators on CBF of airway epithelium. Many drugs and excipients have been shown to affect CBF and mucus clearance. Beta-adrenergic and cholinergic agents stimulate CBF, while fluticasone, some preservatives, and some bacterial toxins inhibit CBF [13,16,18,21,50,58,106,107,108].

**Figure 5:**
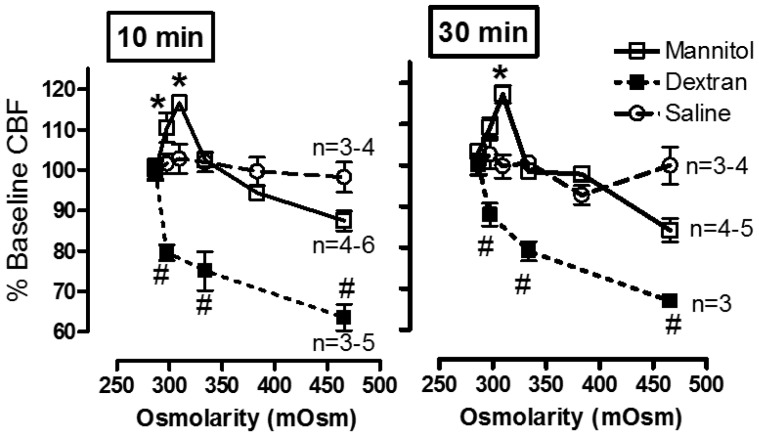
Effect of mannitol, dextran, and saline on CBF of ciliated HBEC. CBF (Hz) was measured from the same sites (10–12 epithelial cell strips) before and after perfusion with mannitol, dextran, or saline for 10 min or 30 min. CBF increased after acute (10 and 30 min) exposure to mannitol and decreased with dextran. No significant change in CBF occurred with hypertonic saline. A fresh batch of HBEC was used per agent. Data shown as % baseline CBF (mean ± SEM). * **#** ANOVA plus Tukey’s test, *p* < 0.01 for 10 min; *p* < 0.05 for 30 min.

- **The effect of exogenous and endogenous mediators on CBF:** Airway cilia function can be modified by various endogenous and exogenous exposures (Figure 4) including infections such as rhinitis, rhinosinusitis [6,76,77], and pneumonia [78]; environmental pollutants [79,80,81]; cigarette smoke and oxidative stress [82,83,84,85], and the rheology of the mucus secretions [75,86,87]. Numerous agents and drugs (Table 1) have been shown to modulate CBF [16,17] with efficient mucociliary transport relying on effective regulation of ciliary beating [11,22]. In vitro, beta-adrenergic and cholinergic agents were demonstrated to stimulate CBF, while fluticasone, some preservatives, anaesthetics, and some bacterial toxins inhibited CBF [16,17,18,19]. In addition, the effect of hyperosmolar agents on the muco-ciliary transport apparatus has been documented [38,75]. In a study of the direct effect of mannitol on the CBF of human bronchial epithelial cells (Figure 5), we demonstrated a direct osmolarity-independent cilio-stimulatory effect, a unique mechanism of action for mannitol, compared to dextran and saline, on ciliary beating [53]. A systematic review of the literature that examines the effect of 229 drugs and other substances on CBF of airway epithelial cell cilia has been published [20].
- **Relationship of inflammation to reduced or variable CBF:** earlier studies in patients with cystic fibrosis have demonstrated a link between airway inflammation and a reduction in CBF [88,89]. Similar links between inflammation and the impaired mucociliary transport rate were investigated with sputum collected from COPD patients [90], and in ciliated nasal epithelial cells from stable bronchiectatic subjects [91]. However, the effects of inflammation on CBF are not always direct and can reflect modifications of epithelial differentiation and proliferation, possibly related to local chronic inflammation. This occurred in nasal airway cells from Rhinitis patients indicating a possible effect of inflammation on epithelial cell integrity and the number of cilia [92]. In addition, a correlation between ciliary activity and ciliary ultrastructure of the nasal mucosa in chronic and recurrent sinusitis was demonstrated [93] and leukotrienes released during inflammation could slow down CBF [94,95]. Bronchial cilia abnormalities worsened with the disease possibly contributing to the impairment of the mucociliary clearance in chronic bronchitis patients [96]. Also infections such as *P. aeruginosa* and *H. influenzae* caused a significant decrease in CBF of human nasal cilia, while staphylococcal products did not [97]. In comparison, RSV infection of human bronchial epithelial cells in culture resulted in ciliostasis and loss of cilia from the cell surface [98]. Resolution of infection and/or inflammation could restore normal cilia function several weeks following upper respiratory viral infection [99] or acute bronchiolitis in infancy [100].
- **Nasal ciliary changes can reflect bronchial changes:** Respiratory cilia from different locations have been studied. Correlation between nasal, tracheal, and bronchial CBF and between mucociliary clearance and CBF were demonstrated [9,61,101,102,103]. In addition, nasal and bronchial cilia were shown to be comparable in function and structure allowing the use of nasal epithelial cells as surrogates of bronchial epithelial cells in airway inflammation studies [12]. Patients with allergic rhinitis and asthma have stronger nasal responsiveness to cold, dry air compared with patients with rhinitis alone, indicating that upper airway responses could represent lower airway responsiveness [104]. However, the use of nasal cilia as representative of tracheal and bronchial cilia for study of respiratory diseases such as asthma or COPD should be done with caution and preferably in the absence of rhinitis and runny nose due to allergies or active respiratory infections as these directly influence the differentiation and proliferation of nasal cilia and could negate their use as surrogates of tracheal and bronchial cilia. Evidence for this restriction has been demonstrated when intermittent allergic rhinitis was associated with decreased expression of anti-inflammatory genes in nasal fluids obtained from intermittent allergic rhinitis patients [105]. Also, when compared to controls, rhinitis patients exhibited lower percentages of ciliated cells and higher percentages of goblet and basal cells in nasal epithelial brushings related to local chronic inflammation [92]. In addition, respiratory viruses lead to ciliary defects in nasal epithelium of children with recovery occurring within 2–10 weeks after infection [99] and 13–17 weeks following acute bronchiolitis in infancy [100]. All these studies stress the need to study ciliated epithelium specimens in the absence of active respiratory infections.

## 4. Pathophysiology of COPD

**Figure 6:**
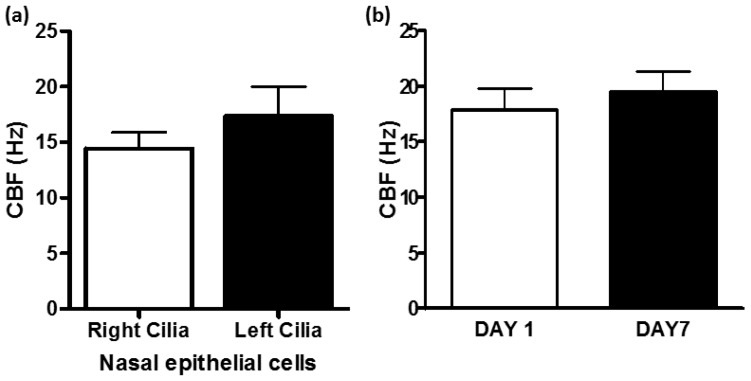
Nasal brushings are non-invasive and reliable to study cilia function and structure. (**a**) CBF (Hz) of ciliated epithelium obtained by brushing the inferior nasal turbinate of healthy subjects: comparison of cilia from the right (*n* = 7) and left (*n* = 3) nasal turbinate. There is no significant difference in CBF of ciliated epithelium obtained from the right and left nasal turbinate; (**b**) CBF (Hz) of ciliated epithelium obtained by brushing of the right inferior nasal turbinate of healthy subjects (*n* = 3). CBF was measured from 8 to 10 sites on the day the sample was obtained (DAY 1), then the sample was stored in the fridge (4 °C) for seven days before another CBF measurement was obtained. Ciliated epithelium can be stored at 4 °C for seven days in Earle’s balanced Salt Solution without any significant change in CBF. Note: Methods as illustrated in Figure 1 and Figure 2. For all epithelial samples, each CBF value is the average CBF measured from 8 to 10 individual sites. Data are expressed as the mean ± SEM (standard error of the mean) of *n* where *n* = number of subjects.

COPD is a leading cause of morbidity and mortality worldwide, and results in a vast economic and social burden that continues to increase. Prevalence and morbidity data greatly underestimate the total burden of COPD because the disease is usually not diagnosed until it is advanced [109]. COPD is a respiratory disorder characterized by progressive, non-reversible airflow limitation, associated with a chronic inflammatory response of the lung to harmful environmental agents (e.g., tobacco smoke, fumes). Inflammatory cells infiltrate the surface epithelium of large airways, contributing to two characteristic symptoms of COPD: cough and sputum production. Persistent inflammation in the small and large airways, and lung parenchyma and its vasculature occurs in COPD patients. The most prominent symptom of COPD is dyspnea, which is largely caused by hyperinflation of the lungs as a result of small airway collapse due to emphysema and narrowing due to fibrosis, so that the alveoli are not able to empty. Hyperinflation reduces exercise tolerance leading to immobility and poor health status [85,109,110,111,112]. Airway inflammation is present even in early disease and persists long after the stimulus (cigarette smoke, pollutants, and noxious gases) is withdrawn [113,114]. In COPD, cilia can be overwhelmed with secretions or they could be abnormal in terms of structure, but we can readily investigate ciliary function. Nasal brushings from COPD patients and healthy subjects are non-invasive and provide a quick method to study ciliary function and structure ex vivo. Brushings can be obtained from the right or left nasal turbinate and can be stored for up to seven days without any significant change in CBF measurements (Figure 6) [49].

## 5. Factors That Can Affect CBF in COPD

**Figure 7:**
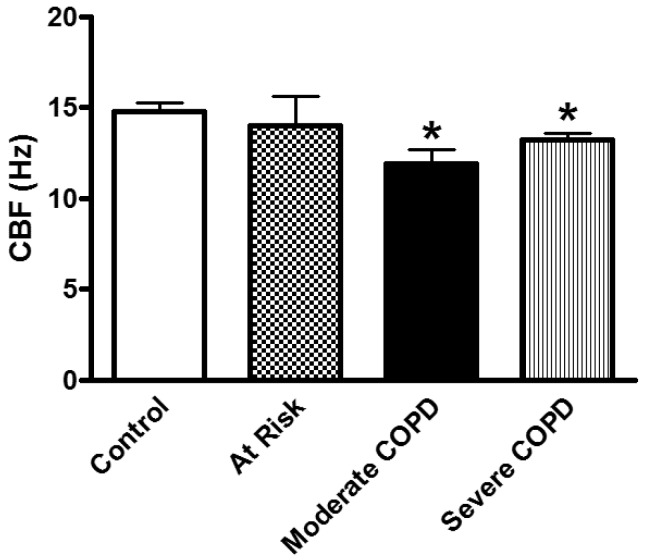
CBF (Hz) of nasal cilia was significantly depressed in cilia obtained from moderate and severe COPD subjects compared to At Risk and Control subjects. CBF (Hz) of ciliated nasal epithelium of Control (*n* = 6), At Risk (cough and phlegm but no COPD) (*n* = 7) and COPD subjects (Moderate, *n* = 5 and Severe, *n* = 7). * *p* = 0.029, Kruskal-Wallis test. For all epithelial samples, each CBF value is the average CBF measured from 10 to 12 individual sites. Data are the mean ± SEM of n where *n* = number of subjects.

**Figure 8:**
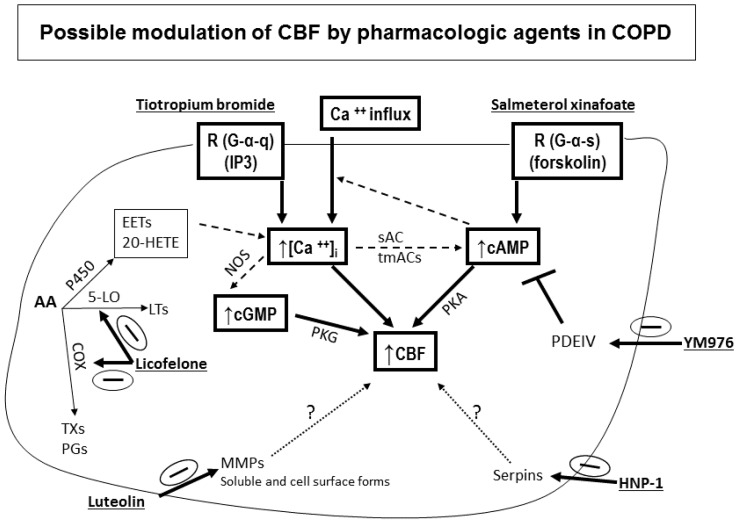
Possible modulation of CBF by pharmacologic agents in COPD. Representative schemes of the possible sites of action of the pharmacologic agents tested on nasal cilia obtained from Control, At Risk, and COPD subjects. Information presented here is derived from the review of signaling pathways involved in mammalian ciliary beating [13], and references in our COPD paper [52] and in this review. Beta agonists cause an immediate cAMP-dependent increase in CBF, followed by a cAMP-dependent increase in intracellular calcium, which in turn increases CBF. Salmeterol (1 µM), a beta-adrenergic agonist, increases CBF in a cAMP-dependent manner via beta two receptors (G-alpha-s coupled R) expressed at the apical membranes of epithelial cells—i.e., acts via increased cAMP, PKA activation, and increased CBF due to phosphorylation of axonemal proteins. Tiotropium Bromide (5 µM) results in muscarinic AchR inhibition (i.e., inhibits muscarinic M3 receptors on parasympathetic postganglionic effector cell junctions) and plays a role in increased intracellular calcium leading to increased CBF; also, increased intracellular calcium activates NOS leading to increased cGMP and increased cGMP-dependent protein kinase and increased CBF. The effect is on a G-alpha-q coupled R and IP3 release which leads to increased calcium influx. Licofelone (10 µM), a dual inhibitor of cyclooxygenase and 5-lipoxygenase pathways—i.e., inhibits 5-LO LTs (LTB4, C4 and D4), prostaglandins, and thromboxanes. Inhibition of both pathways leads to a shift into the metabolism of AA to CYP metabolites (EETs and 20-HETE) which activate influx of calcium into the cell and increased CBF. PKA and PKG activation are also possible. Luteolin (100 µM) inhibits activity of metalloproteinases MMP-2 and MMP-9. MMPs are not expressed in normal healthy tissues but in diseased tissues that are inflamed or undergoing remodeling and repair and MMPs may be compartmentalized in close proximity to the cell surface—i.e., soluble and cell surface forms. YM976 (100 nM) inhibits PDE4 (PDEIV) resulting in increased cAMP and PKA activation and increased CBF. HNP-1 (6 µM), human neutrophil protein-1 inhibits serpins and also acts as an endogenous antimicrobial agent. Note that increased intracellular calcium leads to activation of ca-dependent cyclases (AC1, 3 and 8) which increase cAMP and lead to increased CBF. In summary, whether the agent modulates a receptor (adrenergic/cholinergic), an enzyme (5-LO, COX, PDE4, serpins), or structural components (MMPs soluble and cell surface forms)—i.e., different mechanisms of action—the final outcome is an increased CBF. From the literature, we know that the main second messengers following receptor stimulation are cAMP and calcium and the final step in increased CBF is phosphorylation of axonemal proteins, suggesting a common signaling pathway among all the agents tested. Further investigation of the intracellular signaling pathways is needed. R: receptor; IP3: inositol tris-phosphate; P450: cytochrome P450; 5-LO: 5-lipoxynase; COX: cyclooxygenase; EETs and 20-HETE cytochrome P450 metabolites that result in increased calcium influx into the cell; PDEIV, phosphodiesterase type 4; cAMP, cyclic adenosine monophosphate; cGMP, cyclic guanosine monophosphate; NOS, nitric oxide synthase; PKA, protein kinase A; PKG, cGMP-dependent protein kinase; sAC, soluble adenylyl cyclase; tmACs, transmembrane adenylyl cyclases; Serpins, serine protease inhibitors.

Hurst et al. reported a correlation between the degree of upper and lower airway inflammation in COPD highlighting the significance of studying ciliated nasal epithelial cells in this disease [115]. In addition to inflammation, another process thought to be important in the pathogenesis of COPD is an imbalance of proteinases and antiproteinases in the lung [43]. Roles for defensins, matrix metalloproteinases, and inflammatory mediators have been implicated in COPD [109,116,117,118,119]. Exhaled leukotrienes and prostaglandins are also elevated in COPD [120]. Physiological changes characteristic of the disease include mucus hypersecretion and ciliary dysfunction. To be effective in clearing secretions out of the lung, the mucociliary transport apparatus must exhibit coordinated beating of all ciliated epithelial cells that line the upper and lower respiratory tract. CBF and wave pattern, biologically important measures of epithelial cell function, may be altered during an exacerbation, leading to accumulation of secretions and impairment of beating. We speculate that this impairment may take the form of cilia unable to initiate the effector or return stroke of the beat cycle due to increased mucus viscosity or ineffective transport of mucus due to an uncoupling of the cilia tips from the increased depth of the secretion layer. When CBF and wave pattern are abnormal, electron micrographs of cilia ultrastructure may provide useful functional clues in COPD as was demonstrated for PCD and other respiratory diseases [4,64,121]. Differences in CBF have been documented in diverse patient populations [7,122] and numerous drugs and excipients have been shown to affect CBF and mucociliary clearance (MCC) [16,107,108]. As mentioned earlier in this review, beta-adrenergic and cholinergic agents stimulate CBF, while fluticasone, some preservatives, and some bacterial toxins inhibit CBF [16,17,18,19,21,106,107]. However, we do not know the effect of exacerbations on CBF in epithelial cells of COPD patients. Structure-function studies have helped identify the ciliary targets that participate in regulating ciliary beating; however, our understanding of such regulation is far from complete [13]. The ultrastructure of cilia is known [23,24] and correlations between the structure and function of cilia [4,22,25,26,27] have been documented. However, more research is needed to investigate the role of cilia in COPD. An earlier study of nasal CBF in stable COPD found that baseline CBF was reduced by 25% compared to healthy controls and that salmeterol produced a significant increase in CBF [78]. We recently found that CBF of nasal epithelial cell cilia is depressed in subjects with moderate and severe COPD (Figure 7) and that salmeterol could stimulate CBF in cilia from these subjects [52]. Additionally, we demonstrated that pharmacologic agents with various mechanisms of action could modulate CBF of nasal cilia obtained from COPD patients and that a thorough investigation of the various signaling pathways involved in cilia function is still needed [52]. Here we summarize the possible signaling pathways that could be involved in cilia function based on the literature and what we found in nasal cilia from COPD patients (Figure 8). Please note that this is only a suggested starting point for investigating cilia function in COPD. More investigations are needed to fully understand the regulation of cilia function in COPD.

- **Effect of smoking:** Mucociliary clearance has been demonstrated to be affected in smoking [123,124] and while smoke caused cilia loss in bovine lungs studied in vitro [125], ciliary function has been shown to be normal in smoking asymptomatic subjects [64]. When investigating the ultrastructure (TEM) of bronchial epithelial cell cilia, significant ciliary abnormalities in smokers and ex-smokers compared to non-smokers and controls have been observed with the higher percentage of non-specific ciliary abnormalities associated with chronic tobacco smoke in those with chronic sputum production [126], a characteristic symptom of COPD [109,127]. Earlier studies into the effect of smoking on cilia abundance have yielded conflicting results. Rankin et al. (2007) observed a smoking-related difference in cilia abundance in explanted human bronchial tissue [128] similar to that previously described in bronchial [129] and tracheal explants [130]. Other studies, into cilia abnormalities in asymptomatic smokers and nonsmokers, have yielded conflicting results with no difference between asymptomatic smokers and nonsmokers observed in nasal brushings [7,55]. In contrast, Rutland et al. (1983) and Fox et al. (1983), studied cilia ultrastructure in nasal brushings and reported significantly more abnormal cilia in smokers than in non-smokers [131,132]. In a study of bronchial epithelium from normal smokers and matched nonsmokers, the small airway samples (10th to 12th order) had a higher proportion of ciliated cells compared to samples from large (second to third order) bronchi, and smoking caused a decline in the proportion of ciliated cells at both sites [46]. Another study of bronchial biopsies suggested that smoking-associated shorter airway epithelial cilia could play a role in the pathogenesis of smoking-induced lung disease [48]. Cigarette smoke exposure of C57BL/6 mice for 6–12 month was associated with slowed ciliary motility, decreased ciliated cell numbers, and an impaired ciliary response to beta-agonists [133]. In recent studies, exposure of the human epithelium in vitro to cigarette smoke extracts resulted in a broad suppression of genes involved in ciliogenesis [134], and evaluations of cilia lengths from bronchial biopsies or brushings of healthy smokers and nonsmokers showed that smoking is associated with shorter cilia [48,135]. In the large and small airway epithelium, cilia were significantly shorter in COPD smokers compared to healthy smokers and nonsmokers. However, when investigating the gene expression data of the intraflagellar transport system of cilia (which regulates cilia length), some IFT genes were downregulated in smokers but not in COPD smokers compared to healthy smokers [135]. In our COPD study, nasal CBF was significantly depressed in cilia obtained from moderate and severe COPD subjects compared to At Risk and Control subjects (Figure 7). This significant decline in CBF was not due to a difference in the quality of samples (abundance of cilia), or to cell size, cilia length, or cilia coordination. In addition, since At Risk, and COPD (Moderate, Severe) subjects were smokers/ex-smokers with minimum of 32 pack years, the reduced CBF in COPD cilia only could not be attributed just to the effect of cigarette smoke [52]. In addition, various agents could modulate in vitro cilia function of COPD patients confirming a complicated role for cilia in COPD [52] (Figure 8). We also demonstrated that mannitol has a direct effect on CBF of bronchial cilia [53]. Additionally, airway surface properties (hydration, viscosity) can modulate cilia function in vivo as demonstrated in studies of inhaled mannitol in lung disease patients [38,136,137]. This indicates that further studies of the function and ultrastructure of cilia in COPD are needed.

## 6. Factors That Can Affect CBF in Asthma

When investigating the ultrastructure of bronchial mucosa in lung biopsies of children with asthma, the luminal surfaces of ciliated cells showed cytoplasmic blebs and abnormal cilia [138]. Application of Sputum from asthma patients to frogs’ isolated palates to determine the effect on CBF resulted in ciliostasis which disappeared with clinical improvement [139,140]. In comparison, samples obtained from normal volunteers and from most patients, excluding those with asthma, had no effect on ciliary beating. The inhibitory effect on CBF was independent of medications used and was shown in atopic and intrinsic types of asthmatic patients. In addition, the effect was not pH dependent or related to the degree of eosinophilia [140]. Additionally, tracheal mucus velocity (TMV) was significantly less (6.3 ± 2.3 mm per min, mean ± SD, *n* = 6) in asymptomatic asthmatic patients than in normal subjects (11.6 ± 3.6 mm per min, *n* = 7) [141], and tracheal mucociliary transport rates were decreased in patients with allergic asthma [142,143]. Mucociliary function in the airways of patients with bronchial asthma showed abnormalities [144]. Additionally, inhalation of hypertonic saline aerosol or dry-powder mannitol increased mucociliary clearance in asthmatics [145,146], and inhaled steroids restored most of the ciliated surfaces of bronchial epithelial cells [147,148,149]. Therefore, structural and functional ciliary abnormalities have been observed in asthma. Further investigation of cilia structure and function in asthma is needed.

## 7. Summary and Conclusions

COPD is a respiratory disease characterized by airflow limitation that is not fully reversible, is usually progressive, and associated with an abnormal inflammatory response of the lungs to noxious particles or gases. Cigarette smoke is the primary risk factor for COPD. Patients with a smoking history presenting with cough and sputum production, exertional dyspnea, and frequent respiratory infections are likely to be diagnosed with COPD. Exacerbations (whether infectious -bacterial/viral- or non-infectious—second hand smoke/pollution) contribute to the progression of disease severity in COPD. Repeated exacerbations may result in repeated injuries to the airway epithelium and if frequent, could shorten the time available for epithelial repair, resulting in further damage to the integrity of the epithelium due to ongoing inflammation. Inflammation due to exacerbation could alter CBF through mediator release from the inflammatory cells and/or the epithelium or by contributing to cilia damage, epithelial cell loss, and damage to the basement membrane. In addition, the ciliated epithelial cells can spread and transdifferentiate into distinct epithelial cell types to repair the airway epithelium after injury, and this process could be modified in COPD and asthma. Ciliary beating of epithelial cells constitutes an important part of the mucociliary transport apparatus. We demonstrated a significant decrease in CBF of COPD patients classified in the GOLD 2–4 categories implying that impaired ciliary function can impact mucociliary clearance in COPD, potentially contributing to retention of secretions and infection. Pharmacologic agents with different mechanisms of action can similarly increase CBF of nasal cilia of COPD subjects compared to healthy subjects. Further investigation of the signaling pathways involved is needed. Additionally, the acute and prolonged effects of mannitol on CBF of HBEC suggest a unique mechanism of action for mannitol on ciliary function. Continued investigation of the function and structure of airway epithelial cilia in COPD could lead to a better understanding of the mechanisms of disease and to improved therapies. The investigation of epithelial repair and the role of cilia abnormalities in the initiation and progression of COPD is still needed. Specifically, aspects of ciliary function and structure that may affect COPD, namely CBF, coordinated ciliary beating, the ratio of ciliated to non-ciliated areas, and the susceptibility of the cilia to intrinsic and extrinsic agents that modify their rate and quality of motion should be further investigated. Additionally, fewer investigations on the above parameters have been published in patients with asthma including the effects of chronic treatments (e.g., steroids) on ciliary function and structure. Finally, genotypes and how these can influence phenotypes and epithelial cell cilia function and structure should be taken into consideration in future investigations. We believe that the use of models of human airway mucosa is very much needed in respiratory research and will generate answers relevant to the role of cilia in human lung diseases such as COPD and asthma. Use of primary human cells in these models is essential since these cells will mimic more closely the in vivo conditions and allow further investigation of epithelial cell cilia in lung disease.

## Figures and Tables

**Table 1:** Summary of Modulators of CBF *.

Agents/Factors that Increase CBF	Agents/Factors that Decrease CBF	Agents/Factors that Have No Effect
- PKA and PKG activation ↑ CBF - Osmolar agents modulating mucus layer (dextran, mannitol, saline) - mannitol (direct effect on cilia) - Bitter compounds (denatonium and thujone) via T2R4 receptors - Ach (directly)-role for Ca^++^ and M3 receptors - Nicotine (direct effect) - Substance P - Theophylline inhibits PDE_4_ leading to increased cAMP - Inflammatory agents: PGE_1_, PGE_2_, LTD_4_, LTC_4_ - Beta2-adrenergic agonists: Salmeterol and salbutamol via PKA activation and ↑ cAMP - ATP - NO - Hyaluronic acid - Relaxin (↑ cAMP, increases PKA) - Temperature rise: 22 °C to 37 °C - Increased intracellular pH - Increased intracellular bicarbonate - Radiation - Resection of airway (trachea)	- PKC activation ↓ CBF - Fluticasone - Preservatives - Bacterial toxins: *P. aeruginosa* LPS, *H. influenza* LPS - RSV infections - Anesthetics; bipivicaine: ciliotoxic, irreversible - Some allergies reduce CBF: allergic rhinitis - Anti-allergy drugs vary—some increase and some decrease CBF - Hog barn suppresses cilia response to stimulation - Cotinine (metabolite of nicotine)	- Propranolol - Midazolam - Ribavirin - Histamine - PGF_2_-alpha - Staphylococcus - Some allergies

* Summarized from references in this review.
